# Dengue virus at the landscape scale in Cambodia: Linking climate and land use dynamics with Dengue ecology in Southeast Asia

**DOI:** 10.21203/rs.3.rs-10085402/v1

**Published:** 2026-07-14

**Authors:** Sahina SIDHIK, Kimly HENG, Kimsear NOV, Sokchea LAY, Sreymom KEN, Sopheak SORN, Leangyi HENG, Y Phalla, Anavaj SAKUNTABHAI, Sowath LY, Veasna DUONG, Claude FLAMAND, Tineke CANTAERT, Sébastien BOYER

**Affiliations:** Institut Pasteur du Cambodge; Institut Pasteur du Cambodge; Institut Pasteur du Cambodge; Institut Pasteur du Cambodge; Institut Pasteur du Cambodge; Institut Pasteur du Cambodge; Institut Pasteur du Cambodge; Institut Pasteur du Cambodge; Institut Pasteur; Institut Pasteur du Cambodge; Institut Pasteur du Cambodge; Institut Pasteur du Cambodge; Institut Pasteur du Cambodge; Institut Pasteur du Cambodge

**Keywords:** Aedes, climate variability, dengue transmission, land use dynamics, seroprevalence, vector ecology

## Abstract

Dengue remains a major public health challenge in Cambodia, where transmission dynamics are driven by interactions among human infections, *Aedes* vector populations, land use and climatic variability. Hence, longitudinal evidence linking laboratory-confirmed dengue infections with entomological and environmental indicators remains limited. This study aimed to investigate the dengue incidence and *Aedes* vector populations in relation to climate and land use dynamics. Adult *Aedes* mosquitoes have been collected across six major land-use types using BG-Sentinel traps every 2 months during 4 years. In parallel, a prospective householdbased cohort study was conducted to enrolling individuals residing in trap-associated households. Blood samples have been collected twice annually and tested for anti-dengue virus (DENV) IgM and IgG antibodies using in-house ELISA assays. Entomological, serological, and climatic data were integrated and analyzed using different models to assess immediate and delayed associations between vector density, climate variables and dengue cases. *Aedes aegypti* was consistently more abundant across all habitats, particularly in urban and wetland landscapes. *Aedes albopictus* predominated in wooded and residential areas, reflecting a preference for shaded habitats, though low dry-season abundance limited broader ecological inference. Among approximately 70 individuals with paired samples, ~ 17% were dengue-positive, corresponding to an estimated incidence of 8.6 infections per 100 person-years. *Aedes aegypti* abundance was significantly associated with dengue incidence, whereas *Ae. albopictus* showed no significant relationship. Distributed lag analyses further identified delayed effects of *Ae. aegypti* density on dengue risk within 0–4 weeks. This integrated study highlights the combined influence of human infection patterns, vector ecology, climatic variability, and landscape context on dengue transmission in central Cambodia, supporting to improve dengue risk assessment and location-specific vector control interventions.

## 1. Introduction

Dengue virus (DENV) is a prevalent arboviral pathogen comprising four antigenically and genetically distinct serotypes (DENV1–4) [1, 2]. Southeast Asia represents one of the most dengue-endemic regions globally, accounting for a substantial proportion of the world’s dengue cases [3, 4]. The region’s rapid urbanization, dense populations, and climate variability have created complex eco-epidemiological settings that sustain vector breeding and transmission. Countries such as Thailand, Vietnam, Laos and the Philippines report similar seasonal epidemics, reflecting common climatic drivers—particularly monsoon-associated rainfall, humidity, and temperature cycles—that amplify *Aedes* mosquito abundance and dengue transmission [5, 6]. Yet, within this shared regional context, localized land use practices and environmental modifications generate heterogeneous risk patterns. Understanding Cambodia’s dengue ecology within this wider Southeast Asian setting provides critical insights into both the similarities and the unique ecological pressures shaping vector populations and disease dynamics across the region.

It is a major and escalating public health concern in tropical and subtropical regions, with Cambodia experiencing persistent and widespread outbreaks [7]. Transmitted primarily by *Aedes aegypti* and *Ae. albopictus*, dengue infections display distinct seasonal patterns, typically intensifying during the rainy season when vector populations expand [8, 9]. Recent data reflect ongoing challenges, Cambodia experienced a severe dengue outbreak in 2025, with 63,016 reported cases and 79 deaths as of December 31, 2025, marking a 232% increase from 2024 [10]. Although annual case numbers fluctuate, dengue transmission remains endemic, with children accounting for a substantial proportion of severe cases and fatalities, often associated with delayed healthcare access [11, 7]. Sustained transmission across both urban and rural settings underscores the need for a better understanding of dengue transmission dynamics to support evidence-based surveillance and targeted public health interventions [12, 2].

The abundance and distribution of *Ae. aegypti* and *Ae. albopictus* are primarily driven by meteorological and climatic factors, while environmental and land-use features further influence their breeding sites. Land use changes, such as urban expansion, deforestation, rice-field increase and the development of wetland or riverine areas, can create suitable breeding habitats and modify local microclimates that favor mosquito proliferation [13, 14]. In Cambodia, rapid socio-economic transformations have altered land cover over the past decades, yet the effects of these changes on dengue vector ecology remain poorly characterized [15]. In addition, *Ae. aegypti* and *Ae. albopictus* exhibit ecological differences in habitat preference and behavior, with inferences for virus transmission across diverse landscapes. These differences have epidemiological consequences, as landscape-driven variation in vector composition and density may translate into heterogeneous patterns of human dengue risk, yet this link remains poorly identified in Cambodia [16, 17]

Longitudinal entomological surveillance combined with human dengue monitoring helps identify time delays between mosquito abundance, climatic factors, and dengue cases, improving understanding of disease transmission [18, 19]. Spatiotemporal modeling approaches can then be used to support risk mapping, early warning systems and targeted public health interventions [20, 21]. This study advances our understanding of dengue ecology in Cambodia by examining how climate and land use jointly influence vector populations across urban, residential, wetland, wooded, riverine and rice-field areas. We applied time-series analyses and climate–landscape models to characterize seasonal and spatial dynamics of vector abundance and to link these patterns with serologically confirmed human dengue infections.

## 2. Materials & Methods

### a. Study area

**Figure 1:**
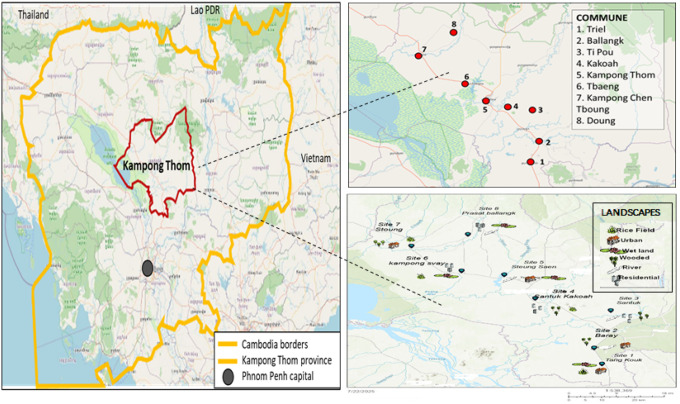
Location map of the study area representing six landscape types. Each site is georeferenced and visually distinguished according to its respective landscape category.

This study was conducted in Kampong Thom province, central Cambodia (10°–12°N, 104°–105°E), featuring a tropical monsoon climate with average annual rainfall of 1,200–2,000 mm and distinct rainy (June-October) and dry (November-April) seasons. Eight districts—Tang Kouk, Baray, Santuk, Santuk Kakoah, Steung Saen, Kampong Svay, Stoung, and Prasat Ballangk—were selected based on accessibility, prior vector surveillance and representation of diverse landscapes relevant to *Aedes* mosquito habitats (Fig. 1). These sites represent a gradient of six major land use and land cover types: Urban, Residential, Rice-field, Wetland, Riverine, and Wooded, classified using high-resolution satellite imagery. Mosquito sampling locations were assigned to predominant landscape categories by applying a 500-meter buffer around trap sites (**Supplementary Fig. 1**). Across the eight districts, a total of 24 sentinel sites were established, with a target of four sites per landscape type (Table 1).

### b. Mosquito sampling and identification

Adult mosquito surveillance for *Ae. aegypti* and *Ae. albopictus* were conducted from January 2021 to December 2024 across sentinel sites in Kampong Thom province. In 2021, sampling was performed monthly in both indoor and outdoor environments at all sentinel sites, while from 2022 to 2024, collections were carried out bimonthly (once every two months) following the same protocol. Adult mosquitoes were collected using BG-Sentinel traps (Biogents AG, Regensburg, Germany) equipped with BG-Lure attractants. At each sampling site, traps were operated continuously for three consecutive days, with battery replacement every 24 hours to ensure consistent trapping efficiency. Captured mosquitoes were transported to the laboratory and stored at − 20°C until identification. Specimens were morphologically identified to species level using standard taxonomic keys [22, 23]. Samples exhibiting ambiguous morphological characteristics were further confirmed through molecular identification based on Polymerase chain reaction (PCR) amplification of the internal transcribed spacer 2 (ITS2) and cytochrome oxidase subunit I (COI) gene regions, following previously described protocols [24]. PCR products were purified and sequenced, and species identity was validated through BLAST analysis against reference sequences available in the GenBank database. All field and laboratory procedures adhered to the World Health Organization’s entomological surveillance guidelines [23].

### c. Human sample collection

From 2022 to 2024, blood samples were collected from 89 individuals residing in selected households where mosquito traps were installed. Sampling was conducted twice annually within a longitudinal follow-up framework (n = 6). Participants were contacted every two months throughout the study period to record hospitalization events and the occurrence of dengue-like clinical signs and symptoms. The total number of participants enrolled, the number of paired serum samples obtained, and the follow-up completion rate are reported in the results section. Blood samples were transported to the virology unit at the Institut Pasteur du Cambodge (IPC), where sera were separated and stored at −80°C until serological analyses were performed.

Ethical approval for the clinical study was obtained from the National Ethics Committee for Health Research of Cambodia (NECHR nr 2020 – 214). Written informed consent was obtained from all participants or from the legal guardians of participants under 16 years of age prior to inclusion in the study.

### d. IgM detection by MAC-ELISA and IgG detection by indirect ELISA

Anti-DENV IgM antibodies (Abs) in human serum samples were detected using an in-house capture ELISA, as described previously [25]. Briefly, 96-well plates were coated with a 100 μL of 1 μg/mL goat anti-human IgM Abs (Sigma Aldrich) for 4 hours at room temperature (RT). After blocking for 30 minutes, a 100μL of pre-diluted sera was added, follow by adding mouse sucrose-acetone extracted DENV1, DENV2, and DENV3. The plates were incubated overnight at 4°C. After washing, DENV mouse hyper-immune ascites was added and incubated at 37°C for 1 hour. Subsequently, peroxidase conjugated Fab anti-mouse IgG H + L Abs were added, followed by KPL ABTS (SeraCare life science, USA). Optical density (OD) was read at wavelength 620nm after 10 minutes incubation. Anti-DENV IgG in human samples were detected using in-house indirect ELISA as previously described [25]. Briefly, a mixture of the four serotypes of DENV cell culture lysate antigen was coated onto 96-well plates and incubated at 4°C overnight. The plates were then blocked with 5% skimmed milk for 30 minutes at RT, and 100μL of pre-diluted sera was added. After washing, goat peroxidase-conjugated anti-human IgG Abs (Sigma Aldrich) were added. Following a 1hour incubation, KPL ABTS (SeraCare life science, USA) was added, and OD was read at wavelength 620nm after 10 minutes incubation. Inter- and intra-precision of the different assays was assessed using one negative control, one weakly positive control and one strongly positive control. A sample was defined positive when the OD value was higher or equal 0.1 for IgM and higher or equal 0.05 for IgG.

### e. DENV infection

DENV infection cases were defined based on paired serum samples collected at two time points approximately six months apart. Recent infection was identified by (1) IgM seroconversion, defined as IgM-positive or a transition from IgM-negative to IgM-positive status between paired samples using an in-house DENV IgM capture ELISA, or (2) IgG seroconversion (negative to positive) or a ≥ 4-fold increase in anti-DENV IgG antibody titres between paired samples, as measured by an in-house ELISA [25]. To minimize potential cross-reactivity with other co-circulating flaviviruses, serological results were interpreted conservatively, requiring clear seroconversion or significant titre increases between paired samples rather than single time-point positivity. In addition, assay performance and antigen specificity followed previously validated protocols [25].

### f. Spatial distribution of Dengue cases and vector populations

The association between vector density and dengue incidence was assessed through a sequential analytical framework. First, a temporal trend analysis was performed by plotting the monthly time series of *Ae. aegypti* density, *Ae. albopictus* density, and dengue case counts. Subsequently, Pearson correlation coefficients and simple linear regression models were used as an initial descriptive assessment of the concurrent associations between the density of each *Aedes* species and the number of concurrent dengue cases. Finally, to investigate potentially delayed and nonlinear effects of mosquito density on dengue risk, Distributed Lag Nonlinear Models (DLNM) were fitted using the dlnm package in R [26]. The exposure–response relationship (mosquito density) was modelled using a natural cubic spline with X degrees of freedom, while the lag–response dimension was also modelled using a natural cubic spline with Y degrees of freedom over a lag period of 0–12 weeks. Dengue case counts were modelled using a quasi-Poisson regression framework to account for overdispersion in the outcome data. Model specification was guided by minimization of the Akaike Information Criterion (AIC) alongside assessment of model fit and biological interpretability. Cumulative and lag-specific relative risks (RR) with 95% confidence intervals were estimated across varying levels of mosquito density and lag periods, allowing quantification of both immediate and delayed effects of vector abundance on dengue incidence.

### g. Meteorological data collection and climate trend analysis

Monthly climatic variables—including mean temperature (°C), relative humidity (%), total rainfall (mm), and mean wind speed (m/s)—were obtained from the NASA POWER (Prediction of Worldwide Energy Resources) Data Access Viewer (DAV). To facilitate seasonal analysis, months was classified according to Cambodia’s climate into wet season (June to October, characterized by high rainfall and humidity) and dry season (November to April, with lower precipitation and reduced humidity). Temporal and inter-annual trends in climatic variables were then analyzed to provide an environmental framework for *Aedes* mosquito ecology. (**Supplementary Fig. 2**)

### h. Mosquito-climate modeling and Time series analysis

Mosquito–climate relationships were assessed using Generalized Additive Mixed Models (GAMMs), with mosquito counts as the response variable and monthly climate variables as predictors. Although GAMMs accounted for clustering at the sentinel site level through random effects, residual temporal autocorrelation and unmeasured time-varying confounders may still influence mosquito abundance dynamics. In addition, the relatively limited number of observations per site and the overall temporal resolution of the dataset may constrain the precision of estimated nonlinear climatic effects. These limitations should be considered when interpreting the strength and generalizability of the observed climate–mosquito associations. Seasonal-Trend Decomposition based on Loess (STL) was applied to the mosquito count data and climate variables. To forecast short-term mosquito abundance, the Holt-Winters exponential smoothing method was implemented. All, time series analyses were conducted separately for *Ae. aegypti* and *Ae. albopictus*.

### i. Statistical tools and software

All statistical and spatial analyses were conducted using R (version 4.5.1), QGIS (version 3.36) and ArcGIS (ArcMap 10.8.2). Data preprocessing, modeling, and visualization were primarily implemented in R, while spatial data integration and map generation were performed using QGIS and ArcGIS. All scripts were run on a windows-based system, and results were reproducible across platforms.

## 3. Results

### a. Dengue vector abundance by landscape and season

**Figure 2:**
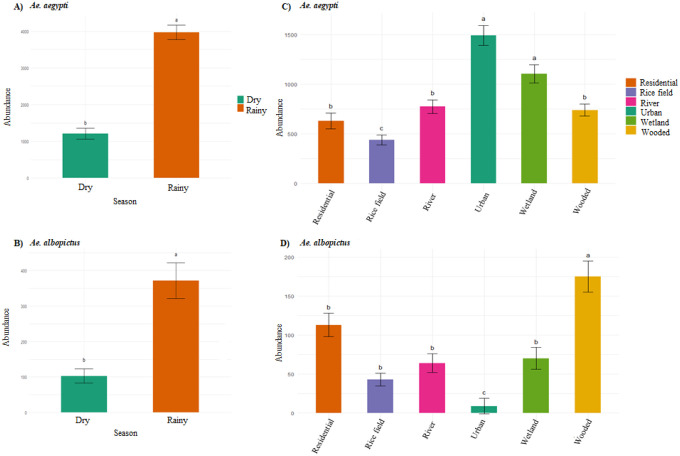
Seasonal and landscape-associated variation in *Aedes aegypti* and *Ae. albopictus* abundance across study sites in Cambodia. (A–B) Seasonal variation in *Ae. aegypti* and *Ae. albopictus* abundance. (C–D) Distribution of *Ae. aegypti* and *Ae. albopictus* across different landscape types.

The abundance and distribution of *Ae. aegypti* and *Ae. albopictus* varied significantly across landscape types, seasons, and sampling years in Kampong Thom province (Table 2). Among the six landscape categories, urban areas (1,487 individuals; average 40 mosquitoes per house) and wetlands (1,103; 19 per house), supported the highest *Ae. aegypti* abundance, followed by wooded areas (739; 16 per house), riverine landscapes (773; 15 per house), residential areas (629; 12 per house), and rice-field landscapes (437; 9 per house). In contrast, *Ae. albopictus* was significantly most abundant in wooded (176; 3 per house) than in wetland and (126; 3 per house), compared to residential habitats (113; 2 per house), and more present than in riverine (64; 1 per house), rice-field (43; 1 per house) and urban areas (9; <1 per house) (Fig. 2). Seasonal analysis revealed that *Ae. aegypti* were significantly 3.5-fold higher during the rainy season compared to the dry season (median 140 vs. 40 per month). *Aedes albopictus* exhibited an increase of 2.2-fold (median 18 vs. 8 per month), indicating a more pronounced seasonal amplification. GAM analyses confirmed significant strong positive correlations of *Ae. aegypti* with urban, wetland, riverine, and wooded landscapes and a negative correlation with rice fields (p < 0.01), while *Ae. albopictus* was significantly correlated with wooded areas (p < 0.001) but negatively correlated with urban, rice-field, riverine, and wetland habitats (p < 0.01) (**Supplementary Table 1**).

### b. Spatial and temporal association between dengue cases and vector populations

**Figure 3:**
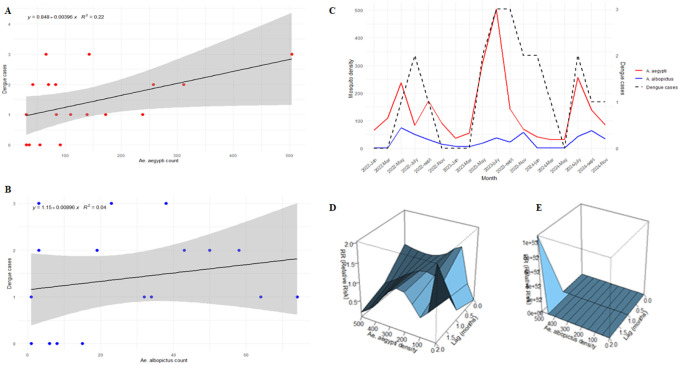
Spatial and temporal associations between dengue incidence and *Aedes* vector abundance. (A–B) Relationships between *Ae. aegypti* and *Ae. albopictus* densities and dengue incidence. (C) Temporal trends in *Aedes* abundance and dengue cases from 2022–2024. (D–E) Distributed lag nonlinear model (DLNM) showing the effects of *Ae. aegypti* and *Ae. albopictus* abundance on dengue risk.

Blood samples have been collected from 89 individuals residing in 24 households where mosquito traps were simultaneously installed, within a longitudinal follow-up framework conducted between 2022 and 2024. Sampling was performed twice annually, generating paired serum samples for serological assessment of dengue virus exposure. To ensure temporal alignment with epidemiological data, entomological and dengue surveillance datasets were harmonized into 18 monthly observation units, each representing mean monthly *Aedes* spp. abundance across sentinel sites paired with corresponding provincial dengue case counts (Fig. 3A–B). Among approximately 70 individuals with paired samples, ~ 12 (≈ 17%) were positive, corresponding to an estimated incidence of 8.6 infections per 100 person-years over the study period. Notably, infections were spatially heterogeneous, with clustered cases observed in specific urban and residential settings. Temporal trend analysis revealed distinct seasonal fluctuations in *Ae. aegypti* and *Ae. albopictus* densities alongside dengue incidence between 2022 and 2024 (Fig. 3C). Peaks in *Ae. aegypti* abundance closely coincided with increases in dengue cases, particularly during the rainy seasons, whereas *Ae. albopictus* exhibited relatively lower and more stable population levels throughout the study period. Linear regression analysis demonstrated a significant positive association between *Ae. aegypti* density and dengue incidence (R^2^ = 0.22; p < 0.05), supported by a moderate positive correlation (Pearson r = 0.44), indicating its epidemiological relevance in local transmission dynamics. In contrast, *Ae. albopictus* showed no significant association with dengue incidence (R^2^ = 0.04; p > 0.05), with a weak negative correlation (Pearson r = − 0.18), suggesting a limited role in transmission in this setting. The DLNM, further highlighted a delayed but significant effect of *Ae. aegypti* density on dengue risk, with relative risk increasing sharply at higher vector densities, particularly within short lag periods of 0–4 weeks. No meaningful lagged association was observed for *Ae. albopictus*, consistent with its lower abundance and weaker epidemiological linkage (Fig. 3D–E).

### c. Comparative analysis of climatic influences on Ae. aegypti and Ae. albopictus

**Figure 4:**
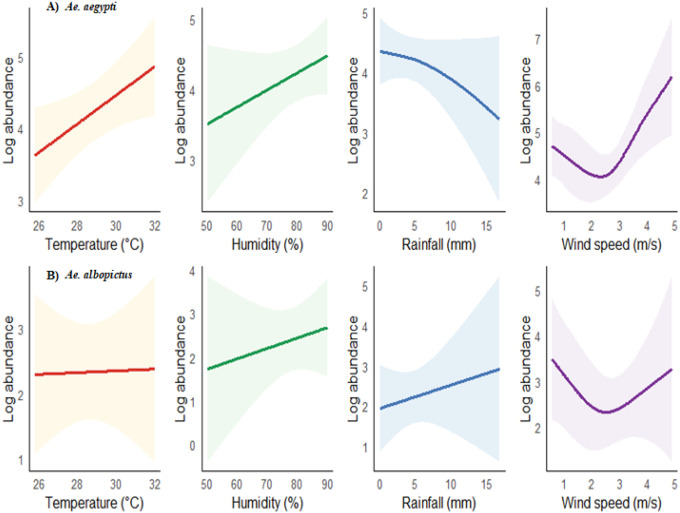
Generalized Additive Model (GAM)–based effects of climatic variables on *Aedes aegypti and Ae. albopictus* mosquito abundance.

GAMs were used to examine the nonlinear effects of climatic variables *on Ae. aegypti* and *Ae. albopictus* abundances across the study period. For *Ae. aegypti*, temperature and humidity showed positive relationships with mosquito abundance, with abundance increasing steadily across the observed temperature range (26–32°C) and relative humidity levels (50–90%). Rainfall displayed a predicted abundance at moderate rainfall but gradually declined with increasing rainfall. Wind speed exhibited a nonlinear response. In contrast, *Ae. albopictus* exhibited comparatively weaker climatic responses. Temperature showed only a minimal effect on abundance, remaining nearly constant across the observed range. Humidity and rainfall demonstrated gradual positive associations, with abundance increasing steadily under wetter conditions. Wind speed also showed a nonlinear relationship, with abundance decreasing at intermediate wind speeds before increasing again at higher wind speeds (Fig. 4). Model diagnostics confirmed the robustness of the climatic GAMs, with residual analyses indicating acceptable linearity, normality, and homoscedasticity assumptions. No influential observations were detected (Cook’s distance < 0.5), supporting the reliability of the climatic inferences for both mosquito species (**Supplementary Fig. 3**).

### d. Time-series analysis and forecasting of Aedes mosquito abundance

**Figure 5:**
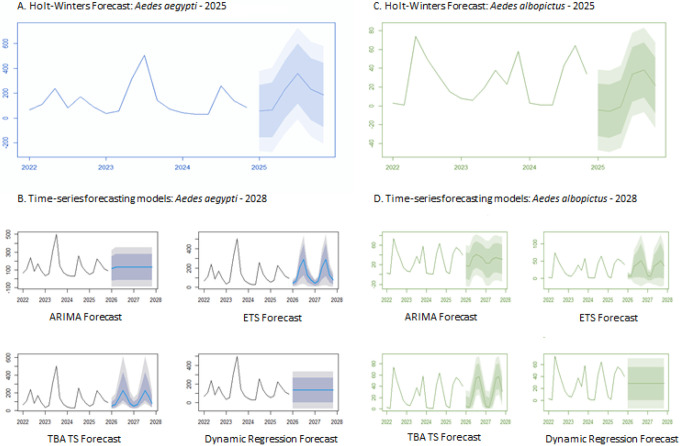
Temporal decomposition and forecasting of *Aedes aegypti* and *Ae. albopictus* abundance from 2022–2028. (A, C) Seasonal–trend decomposition using LOESS (STL) illustrating temporal patterns in *Ae. aegypti* and *Ae. albopictus* abundance from 2022–2026. (B, D) Holt–Winters exponential smoothing forecasts for *Ae. aegypti* and *Ae. albopictus*, including projected trends to 2028 with 80% and 95% prediction intervals.

Time-series analysis of mosquito abundance from 2022 to 2025 revealed distinct species-specific temporal dynamics. Seasonal–trend decomposition using LOESS (STL) identified a stable annual seasonal cycle. The trend component of *Ae. aegypti* increased from 2022 to mid-2023 and subsequently declined through 2024 and slight upsurge into the 2025 period. In contrast, *Ae. albopictus* showed lower overall abundance but persistent fluctuations throughout the study period, with intermittent peaks from 2022 to 2024 but high rise in 2025 (Fig. 5). For *Ae. aegypti*, the Holt–Winters exponential smoothing model showed a root mean square error (RMSE) of approximately 0.08 and a mean absolute error (MAE) of 0.06, reflecting close agreement between observed and fitted values. The coefficient of determination (R^2^) for in-sample fit was 0.86, indicating strong explanatory performance. For *Ae. albopictus*, model performance was slightly less precise but still robust, with RMSE ≈ 1.25 and MAE ≈ 0.98, reflecting greater variability in abundance dynamics; however, observed values remained within acceptable prediction bounds for most time points. Comparative validation across forecasting approaches (ARIMA, ETS, TBATS, and dynamic regression) showed consistent performance for *Ae. aegypti*, with all models producing similarly low error metrics, supporting stable low-level persistence. Forecasts for *Ae. albopictus* were more variable but remained comparable across models (RMSE range: 1.10–1.40; MAE range: 0.90–1.15) ((Fig. 5).

## 4. Discussion

This study demonstrated clear temporal and ecological links between dengue transmission and *Aedes* vector dynamics. Serological surveillance identified evidence of ongoing dengue exposure, with spatially clustered infections observed in urban settings, suggesting localized transmission hotspots. The moderate positive correlation between *Ae. aegypti* counts and dengue cases aligns with findings from central Thailand and southern Vietnam [27–29]. The dengue risk increased within 2–4 weeks after rising *Ae. aegypti* densities, reflecting the extrinsic incubation period of the virus and survival time. Similar lag periods have been reported in Singapore and Indonesia, where dengue incidence followed *Ae. aegypti* abundance with 3–5-week delays [30, 31]. In contrast, *Ae. albopictus* demonstrated no correlations and no significant lagged effects on dengue incidence. Although *Ae. albopictus* is a competent vector, its lower abundance and preference for non-urban habitats likely limit its epidemiological contribution in this region [32, 33]. Comparable results were obtained in Thailand and Malaysia, where *Ae. albopictus* was described as a secondary vector, primarily sustaining transmission in rural and peri-urban fringes [34, 3].

The preferred distribution of *Ae. aegypti* in urban and wetland habitats compared to rice field, residential, wooded and riverine reflects its well-documented adaptation to human-modified environments rich in artificial containers and consistent water availability. Similar trends have been reported in Phnom Penh (Cambodia), Vientiane (Laos), and Bangkok (Thailand), where *Ae. aegypti* thrives in densely populated neighborhoods with inadequate solid waste management and abundant domestic water storage [35–37]. The highest *Ae. aegypti* abundance in urban areas supports evidence that this species is tightly linked to anthropogenic landscapes [38]. The populations in wetlands may be linked to peri-urban expansion zones where stagnant water, vegetation and human activity overlap, creating ecological niches that sustain both *Aedes* species [39]. By contrast, *Ae. albopictus* was most abundant in wooded sites, aligning with its known preference for shaded, vegetated environments [40, 41]. Its lower representation in urban areas underscores its competitive disadvantage against *Ae. aegypti* in urbanized settings, a pattern observed in Ho Chi Minh City and Kuala Lumpur [42]. Ecological partitioning between the two species, therefore, appears driven by microhabitat suitability, oviposition site preference and interspecific competition, as also noted in Vietnam and Thailand [3].

Both *Aedes* species and dengue case trends exhibited strong seasonal patterns, with abundance peaking during the rainy season (May–November). These results are consistent with earlier studies in Cambodia, in Phnom Penh [43, 9] and in other countries in Southeas Asia [44, 45]. In northeastern Thailand humidity above 75% and moderate rainfall levels enhanced larval survival and adult emergence [46]. Our analysis further supports that moderate rainfall and humidity above 80% create optimal breeding conditions by sustaining water-filled habitats for extended periods [47, 48]. Wind speed showed uneven effects on *Ae. aegypti*, possibly reflecting its influence on adult dispersal and resting behavior, as suggested by studies in Malaysia [42]. Conversely, *Ae. albopictus* showed weaker climatic dependence, suggesting broader ecological tolerance and greater climatic plasticity compared with *Ae. aegypti*, consistent with observations from rural Laos and northern Vietnam [49, 19]. These observations are supported by the time-series analyses revealed contrasting population dynamics between *Ae. aegypti* and *Ae. albopictus*. Forecasting analyses further indicated relatively stable *Ae. aegypti* dynamics but a potential increase in *Ae. albopictus* abundance after 2024, which may influence future vector-borne disease risk.

This study has some limitations. First, dengue exposure was assessed using paired serological samples rather than RT-PCR, limiting precise infection timing and serotype identification while introducing possible cross-reactivity with other flaviviruses. The differing temporal resolutions between serological and entomological datasets also require cautious interpretation of vector–dengue associations and DLNM results. However, this serological approach allowed useful identification of DENV exposure at the population level and remains the most feasible strategy for community-based longitudinal studies in resource-limited settings. Second, the relatively small number of sentinel sites and variable sampling frequency may have reduced statistical power and temporal consistency. Third, climatic data derived from NASA POWER could not capture fine-scale spatial variability across study locations. In addition, the statistical models did not account for site-level repeated measures, and potentially important sociodemographic factors influencing dengue risk were not included. Finally, forecasting analyses were based on only four years of data, and therefore medium-term projections should be considered exploratory.

Our study adds to growing regional evidence that dengue transmission in mainland Southeast Asia is influenced by urbanization gradients and climate variability. Across the Mekong basin, *Ae. aegypti* remains the principal driver of dengue epidemics, while *Ae. albopictus* contributes to sporadic and localized transmission in forested and peri-urban zones [37, 36, 3]. The dominance of *Ae. aegypti* in Kampong Thom’s urban and wetland landscapes echoes results from Phnom Penh and Bangkok, where population density, water storage practices, and inadequate waste disposal maintain persistent breeding opportunities [35, 34]. The strong seasonality and climate dependence of *Aedes* populations also reinforce the importance of early vector control interventions ahead of the monsoon season. Targeted community campaigns focusing on container management and larval source reduction should begin before rainfall onset, as recommended by WHO-SEARO guidelines [50]. In contrast, *Ae. albopictus* control may require vegetation management and residual insecticide treatments in shaded areas. Given that both species respond differently to landscape and climate variables, integrated vector management (IVM) approaches should incorporate spatially tailored strategies.

## Supplementary Material

Supplementary Files

This is a list of supplementary files associated with this preprint. Click to download.

- Supplimentaryfile.docx

## Figures and Tables

**Table 1:** Distribution of major land use and land cover types at mosquito sampling sites in Kampong Thom province, Cambodia

No	District	Commune	Urban	Residential	Wetland	Wooded	River	Rice-field	Total Sites
1	Tang Kouk	Triel	1	0	1	0	0	1	3
2	Baray	Ballangk	1	0	0	1	1	0	3
3	Santuk	Ti pou	0	1	0	1	1	0	3
4	Santuk	Kakoah	0	1	0	1	0	1	3
5	Steung Saen	Kampong Thom	1	0	1	0	1	0	3
6	Kampong Svay	Tbaeng	0	1	1	0	0	1	3
7	Stoung	Kampong Chen Tboung	1	0	0	1	0	1	3
8	Prasat Ballangk	Doung	0	1	1	0	1	0	3
Total per habitat			4	4	4	4	4	4	24

**Table 2:** Distribution of *Aedes aegypti* and *Ae. albopictus* across different landscape types and communes over four sampling years.

Landscape	Commune	No. of houses	*Ae.aegypti* (No./year)				*Ae.albopictus* (No./year)			
			Y-1	Y- 2	Y- 3	Y- 4	Y- 1	Y- 2	Y- 3	Y- 4
Residential	Doung	23	155	30	31	15	0	4	0	0
	Kakaoh	10	185	28	11	21	24	32	16	7
	Tbaeng	17	55	29	9	14	4	13	1	4
	Ti pou	7	29	3	7	7	0	3	1	4
Total	4	57	424	90	58	57	28	52	18	15
			629				113			
Rice field	Kakaoh	12	56	4	1	1	0	5	8	10
	Tbaeng	16	45	11	31	5	1	1	1	1
	Kampong Chen	21	107	25	73	6	1	2	1	5
	Triel	3	37	15	16	4	3	1	1	2
Total	4	52	245	55	121	16	5	9	11	18
			437				43			
River	Ballangk	5	108	63	23	26	11	9	9	8
	Doung	24	102	14	27	22	0	2	1	2
	Kampong Thom	14	210	36	66	54	2	12	0	1
	Ti pou	8	18	3	0	1	0	1	3	3
Total	4	51	438	116	116	103	13	24	13	14
			773				64			
Urban	Ballangk	4	63	5	11	9	1	0	4	0
	Kampong Thom	13	251	238	71	58	1	0	0	0
	Kampong Chen	19	309	42	44	29	0	2	0	1
	Triel	1	177	49	74	57	0	0	0	0
Total	4	37	800	334	200	153	2	2	4	1
			1487				9			
Wetland	Doung	22	219	31	480	19	3	37	34	29
	Kampong Thom	15	65	10	20	29	2	3	0	3
	Tbaeng	18	37	15	12	27	0	0	0	1
	Triel	2	100	13	16	10	3	2	7	2
Total	4	57	421	69	528	85	8	42	41	35
			1103				126			
Wooded	Ballangk	6	60	11	23	35	8	1	18	11
	Kakaoh	11	134	17	47	5	13	30	37	10
	Kampong Chen	20	257	61	25	45	4	2	3	0
	Ti pou	9	9	7	1	2	11	13	7	8
Total	4	46	460	96	96	87	36	46	65	29
			739				176			

## Data Availability

Single-cell RNA-seq data have been deposited at Geo: GSE314883 and are publicly available as of the date of publication.
